# Two-Stage Leaching of PCBs Using Sulfuric and Nitric Acid with the Addition of Hydrogen Peroxide and Ozone

**DOI:** 10.3390/ma17010219

**Published:** 2023-12-30

**Authors:** Magdalena Lisińska, Tomasz Wojtal, Mariola Saternus, Joanna Willner, Martyna Rzelewska-Piekut, Krzysztof Nowacki

**Affiliations:** 1 Zakłady Mechaniczne „WIROMET” S.A., ul. Wyzwolenia 27, 43-190 Mikołów, Poland; magdalena.lisinska04@gmail.com; 2 Faculty of Materials Engineering, Silesian University of Technology, ul. Krasińskiego 8, 40-019 Katowice, Poland; tomasz.wojtal@polsl.pl (T.W.); joanna.willner@polsl.pl (J.W.); krzysztof.nowacki@polsl.pl (K.N.); 3 Institute of Chemical Technology and Engineering, Poznan University of Technology, ul. Berdychowo 4, 60-965 Poznan, Poland; martyna.rzelewska-piekut@put.poznan.pl

**Keywords:** hydrometallurgy, PCB, metal recovery, acids, hydrogen peroxide, ozone

## Abstract

The paper presents the possibility of recovering metals from printed circuit boards (PCBs) of spent mobile phones using the hydrometallurgical method. Two-stage leaching of Cu(II), Fe(III), Sn(IV), Zn(II), Ni(II) and Pb(II) with H_2_SO_4_ (2 and 5 M) and HNO_3_ (2 M) with the addition of H_2_O_2_ (10 and 30%) and O_3_ (9 or 15 g/h) was conducted at various process conditions (temperature—313, 333 and 353 K, time—60, 120, 240, 300 min, type and concentration of leaching agent, type and concentration of oxidant, solid–liquid ratio (S/L)), allowing for a high or total metals leaching rate. The use of two leaching stages allows for the preservation of selectivity, separation and recovery of metals: in the first stage of Fe(III), Sn(IV) and in the second stage of the remaining tested metal ions, i.e., Cu(II), Zn(II), Ni(II) and Pb(II). Removing Fe from the tested PCBs’ material at the beginning of the process eliminates the need to use magnetic methods, the purpose of which is to separate magnetic metal particles (ferrous) from non-magnetic (non-ferrous) particles; these procedures involve high operating costs. Since the leaching of Cu(II) ions with sulfuric(VI) acid practically does not occur (less than 1%), this allows for almost complete transfer of these ions into the solution in the second stage of leaching. Moreover, to speed up the process and not generate too many waste solutions, oxidants in the form of hydrogen peroxide and ozone were used. The best degree of leaching of all tested metal ions was obtained when 2 M sulfuric(VI) acid at 353 K was used in the 1st research stage, and 2 M nitric(V) acid and 9 g/h O_3_ at 298 K in the 2nd stage of leaching, which allowed it to be totally leached 100% of Fe(III), Cu(II), Sn(IV), Zn(II), Ni(II) and 90% Pb(II).

## 1. Introduction

In today’s rapidly developing world of technology, electrical and electronic equipment such as TV sets, computers, printers, mobile phones, liquid crystal display (LCD) monitors and laptops have a shorter lifespan compared to devices from previous years [1]. The lifetime of electronic components, which at the end of the 20th century was 4 to 6 years, was shortened to 2 years in the first decade of the 21st century [2]. There are many reasons for shortening the life of electrical and electronic devices, which include: the ever-lower price of devices, as well as fierce competition between companies that want to manufacture and sell the best products based on advanced technologies [1]. An example is a mobile phone called a smartphone, whose sales have increased more than 12-fold in the last 13 years. According to the statistics, in 2020, around 1.57 billion smartphones were sold worldwide, which is a significant increase compared to 680 million units sold in 2012 [3].

Currently, most electrical and electronic devices, especially mobile phones, contain printed circuit boards (PCBs), which are carriers of many metals, including Cu, Fe, Zn, Sn, Ni, Al, Au, Ag, Pt [4,5]. In terms of material composition, the printed circuit board of a mobile phone contains a number of different elements in its structure in the form of metals, plastics, glass or ceramics. Metals account for 40% of the statistical weight of mobile phone PCBs, ceramics 30% and plastics 30%. Depending on the model, manufacturer, design and age of the phone, the content of these components in the phone may vary. The PCBs can contain more than 50 elements. In addition, metals in PCBs can be present in much higher concentrations than in conventional ore deposits, which makes PCB waste a particularly interesting material for recycling [4,5].

PCB recycling fits perfectly into the currently developing model of a circular economy, a concept aimed at using as much recovered materials as possible and using them for re-production, which will reduce the amount of electronic waste and reduce the amount of extracted raw materials [6]. Recycling of PCBs involves many steps, i.e., a combination of physical, chemical, thermal and metallurgical processes [7,8,9,10,11,12]. Pre-processing of electronic waste is usually performed by manual disassembly [10]. The purpose of disassembly is to remove hazardous substances and separate different materials, i.e., metals, glass, plastics and printed circuit boards. Then, the PCBs are mechanically processed, i.e., crushed or shredded. Grinding is an effective operation, as it allows metals to be released from non-metallic parts. This is a key process because grinding improves the efficiency of subsequent mechanical separation and then affects the effective leaching of metals because the leaching agent then has better access to the metal surface [8,10].

The next stage of PCB recycling is methods of physical separation, used to separate the shredded fraction into non-metallic and metallic [7,8]. Magnetic separation of PCBs is usually used, but this has some problems. One is the agglomeration of particles, which attracts a certain fraction of non-ferrous metals combined with the iron fraction. This leads to low effectiveness of this method. Additionally, magnetic separators are ineffective for crushed PCBs. The main challenge with this separation method is the potential loss of valuable precious metals from the printed circuit boards. From an economic point of view, these procedures may involve high operating costs. An interesting alternative may be to skip this stage. The main methods of recovering metals from PCBs are: pyrometallurgical methods [13,14,15,16,17] and hydrometallurgical methods [17,18,19,20,21,22,23,24,25,26,27,28,29,30,31,32,33]. Pyrometallurgy is a widely used metal recovery group of methods involving mainly the melting of waste materials at high temperatures. Hydrometallurgical techniques largely involve acid leaching. Acid leaching with the addition of oxidants is currently the most popular leaching method and has many advantages, including high leaching rate and fast kinetics. Hydrometallurgical methods are more precise, predictable, easy to control and, above all, have a lower environmental impact compared to pyrometallurgical methods. The disadvantage of these methods, however, is that they are slow, time-consuming, require thorough leaching and use large amounts of various types of chemicals, including solvents [18].

The great interest in the subject of hydrometallurgical processing of electronic waste is evidenced by the current number of available scientific publications, amounting to over 50,000 in the Elsevier database. Table 1 presents effective methods of leaching PCBs and high recovery of Cu and other metals, using acids and the addition of oxidants.

The presented results of the use of acids and oxidants prompted us to undertake our own research in this area. In acidic solutions, H_2_O_2_ is one of the strongest, safe and effective oxidants. On the other hand, studies using ozone as an oxidant for PCB leaching have not been used so far (especially as an additive to HNO_3_). It was used, among others, to increase the efficiency of Cu leaching from chalcopyrite. Additionally, one advantage of using this oxidant is the formation of oxygen as the only reaction by-product during leaching. Therefore, in our research, it was decided to use ozone to leach Cu and other metals from PCBs. The aim of the research was to determine the conditions of the two-stage leaching process of Cu, Fe, Sn, Zn, Ni and Pb from PCBs of spent mobile phones using solutions of sulfuric(VI) and nitric(V) acids with the addition of oxidants (hydrogen peroxide and ozone), which will allow high or complete leaching of the metals.

## 2. Materials and Methods

Spent mobile phones from various manufacturers were used as the research material. Most phones were the older models with a numeric keypad (Nokia E50, Nokia 6230i, Nokia 6010, Motorola C168, Motorola W213, Siemens C45, Samsung GU46, Samsung E3210). Initially, manual disassembly of the waste was carried out, separating individual components such as the housing, battery, screen and other minor components from the PCBs. Then, the PCB material was ground in a cross-beater mill (Retsch SK100, Retsch, Düsseldorf, Germany), equipped with sieves with a diameter of 0.2, 1.0 and 5.0 mm. To free the metals embedded inside the PCBs, several batches of PCB material were milled. Sieves of decreasing diameter were used to finally obtain the particulate material. This material was subjected to sieve analysis using a laboratory shaker with an electromagnetic drive (LpzE-2e, MULTISERW-Morek, Marcyporęba, Poland) in order to obtain a particle size of 0.045–4.0 mm. After a sieve analysis, the five fractions were obtained: <0.045 mm (5.74%), 0.045–0.1 mm (14.61%), 0.1–0.8 mm (59.4%), 0.8–1.0 mm (14.83%) and 1.0–4.0 mm (5.42%). The mean particle size was 0.55 ± 0.4 mm. The greater the degree of fragmentation of the material, the greater the mass transfer surface, and then the material prepared in this way was intended for further research.

The analysis of the elemental composition of the milled fraction (0.045–4.0 mm) was performed three times, using the semi-quantitative method, using the WDXRF-Wavelength Dispersive X-Ray Fluorescence Spectroscopy technique. For this purpose, a Rigaku Primus II X-ray fluorescence spectrometer was used. The percentage content of selected metals in PCBs was as follows: Cu—31.97 ± 0.71, Fe—2.90 ± 0.16, Sn—2.47 ± 0.27, Zn—0.3 ± 0.02, Ni—0.91 ± 0.04, Pb—0.34 ± 0.04, Al—2.97 ± 0.15, Au—0.10 ± 0.05, Ag—0.38 ± 0.10, others—57.66%.

The use of two different mineral acids in two different degrees of leaching allows for the separation of, for example, iron and tin from copper. Therefore, based on the literature review and our own research, the following were selected for the two-stage leaching of metals from the ground fraction of PCBs 0.045–4.0 mm:

- sulfuric(VI) acid as a medium for effective Fe leaching—1st stage of research,
- nitric(V) acid and oxidants (hydrogen peroxide, ozone) for extraction of Cu and other metals from PCBs—2nd stage of research,
- temperature 298–353 K.

Hydrogen peroxide is a safe, effective and relatively inexpensive oxidant used to leach PCBs. The end products of its decomposition are oxygen and water. Similarly, for ozone, its use as an oxidant may be a reasonable alternative that also provides environmental benefits in the leaching process, mainly due to the formation of oxygen as the only by-product of the reaction.

**Figure 1:**
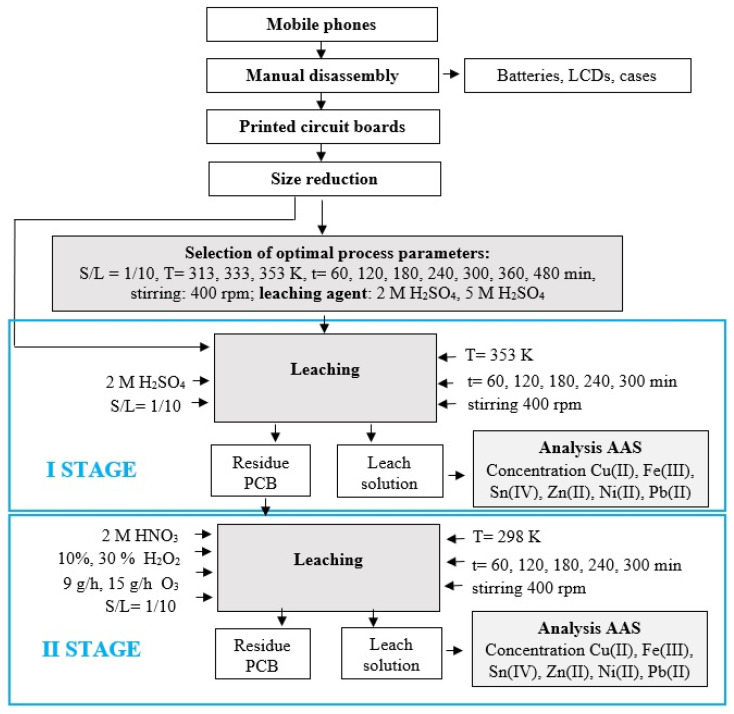
Scheme of two-stage leaching of metals from PCBs with the use of: 1st stage—2 M H_2_SO_4_ at 353 K, 2nd stage—2 M HNO_3_ with the addition of H_2_O_2_ and O_3_ as the oxidants at 298 K.

The two-stage leaching tests were preceded by preliminary metal leaching tests aimed at selecting the optimal parameters for the first stage of the research, which is decisive from the point of view of the conducted research. The performed tests are presented in the form of a block diagram in Figure 1.

Selection of optimal process parameters and concentration of the leaching agent: Experiments were carried out in a glass reactor with a capacity of 600 cm^3^, using the selected leaching agent—2 and 5 M H_2_SO_4_. The temperature of the process was changed in the range from 313 to 353 K, ensuring the mixing of the system with a mechanical stirrer—400 rpm. Leaching was monitored over a period of 60–480 min. The tests were carried out for a constant solid-to-liquid ratio (S/L = 1/10 in g/cm^3^). The concentration of Cu(II), Fe(III), Sn(IV), Zn(II), Ni(II), Pb(II) ions was determined by atomic absorption spectrometry (ContrAA300 spectrometer, Analytik Jena, Jena, Germany). The aim was to select the most convenient leaching parameters at which a high or complete degree of Fe leaching would take place.

Leaching with 2 M H_2_SO_4_ solution (1st stage): For the main tests, 2 M H_2_SO_4_ and a temperature of 353 K were selected as the leaching factor due to the high degree of Fe leaching. In the case of using 5 M H_2_SO_4_, a comparable degree of Fe leaching was obtained in relation to 2 M H_2_SO_4_; however, for economic and ecological reasons, a higher acid concentration was resigned. The first stage consisted of 5 identical leaching tests with the 2 M H_2_SO_4_ solution, S/L = 1/10 g/cm^3^, the mixing speed of the system: 400 rpm, and sampling for the analysis took place after 60, 120, 180, 240, 300 min of leaching. The solid/liquid ratio was selected on the basis of literature reports [27,31,32] and our own research on a laboratory scale.

Leaching with 2 M HNO_3_ solution in the presence of oxidants (2nd stage): For the second stage of the research, 2 M HNO_3_ and the addition of oxidants—10 and 30% hydrogen peroxide and ozone—were selected as the leaching agent. Ozone with a concentration of 0.14 g/dm^3^ and a supply gas volume of 8 dm^3^/min (100% concentration—maximum device capacity of approx. 15 g/h) and 0.07 g/dm^3^ and a supply gas volume of 8 dm^3^/min (concentration 50%—efficiency approx. 9 g/h), was produced using a Korona L20 SPALAB generator, KoronaLab, Piotrków Trybunalski, Poland. The temperature was 298 K. The leaching was also monitored for 60–300 min.

In the second stage of the research, the residue of leached material (ground PCBs after drying) from the first stage was used—5 samples from the 1st stage were leached with 2 M HNO_3_ and oxidants (first sample—HNO_3_, second sample—HNO_3_ + 10% H_2_O_2_, third sample—HNO_3_ + 30% H_2_O_2_, fourth test—HNO_3_ + 50% O_3_, 5th test—HNO_3_ + 100% O_3_). The purpose of the second stage was to leach Cu and the other metals still present in the tested material, obtaining the highest possible degree of extraction of these metals.

## 3. Results

### 3.1. Selection of Optimal Process Parameters and Leaching Agent Concentration

The leaching efficiency of Cu(II) with sulfuric(VI) acid did not exceed 1% in each of the tested cases (2 and 5 M H_2_SO_4_, temperature 313–353 K, 300 min). It is known from the literature data that H_2_SO_4_ is not able to leach copper, and additional oxidants are needed for this, for example, hydrogen peroxide [34,35,36,37,38]. In addition, leaching of Cu with sulfuric(VI) acid in the presence of dissolved oxygen occurs spontaneously and is the main copper oxidation reaction (Equation (1)). Another reaction related to the oxidation of Cu is the reaction with iron(III), in which Fe(III) etches the Cu surface, causing its oxidation (Equation (2)) [39,40].

2 Cu + 2 H_2_SO_4_ + O_2_ → 2 CuSO_4_ + 2 H_2_O (1)

Cu^0^ + 2 Fe^3+^ → Cu^2+^ + 2 Fe^2+^ (2)

The reduction of the available oxygen in the leaching solution severely interferes with the formation of copper(II) sulfate(VI), as shown in Equation (2), which explains the decrease in Cu leaching efficiency. [39,41].

**Figure 2:**
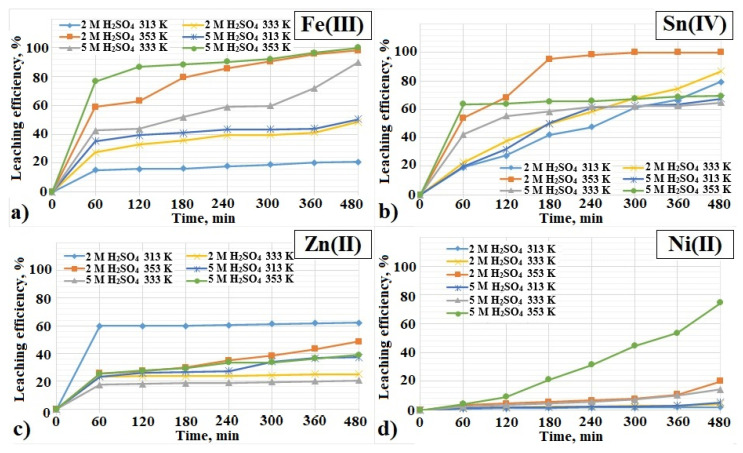
Influence of H_2_SO_4_ concentration on the leaching efficiency of: (**a**) Fe(III), (**b**) Sn(IV), (**c**) Zn(II), (**d**) Ni(II) from PCBs (temperature: 313–353 K, time: 480 min).

The results presented in Figure 2a indicate high leaching of Fe(III) ions with the H_2_SO_4_. An increase in Fe(III) ion leaching is visible from the beginning of the experiment. After 60 min, from about 16 to 76% Fe(III) in the solution was obtained, depending on the concentration of H_2_SO_4_ acid used and the temperature. After 480 min, the highest Fe(III) ion leaching efficiency was 100% using 5 M H_2_SO_4_ (353 K) and 98% using 2 M H_2_SO_4_ (353 K). An increase in the concentration of the leaching agent used resulted in an increase in the degree of Fe(III) leaching. The results of the authors of papers [36,42,43] indicate that H_2_SO_4_ favors the leaching of Fe(III) from PCBs. For further main research (1st stage), 2 M H_2_SO_4_ was selected as an effective Fe(III) leaching agent.

The leaching efficiency of Sn(IV) increased in the initial phase of the process for all considered cases of H_2_SO_4_ acid concentration and applied temperatures (Figure 2b). After 60 min, the Sn(IV) ion leaching result ranged from 20 to 64%. The highest Sn(IV) ion leaching efficiency (100%) was obtained after 480 min, using 2 M H_2_SO_4_ (353 K). On the other hand, 87% of Sn(IV) ions went into solution using the same acid concentration but at a lower temperature (333 K). Using 5 M H_2_SO_4_, the leaching efficiency of Sn(IV) ions after 480 min was about 65–70%. Therefore, with the increase in the concentration of the reagent used, the leaching efficiency of tin(IV) ions did not increase.

Figure 2c shows the leaching efficiency change over time for zinc(II) ions. Already after 60 min, a 60% of leaching of Zn(II) ions was reached using 2 M H_2_SO_4_ (313 K). Over the next 420 min, this figure increased slightly to 62%. Using 5 M H_2_SO_4_, results below 40% Zn(II) ions were obtained. The lowest Zn(II) leaching result, amounting to about 20%, was obtained using 5 M H_2_SO_4_ at 333 K. In the case of Zn(II), the increase in acid concentration also did not affect the degree of Zn(II) leaching. Zinc, unlike copper, may not be present in its metallic form in PCBs, but in the form of zinc(II) oxide and zinc(II) sulfide, which are used as semiconductor elements in electronic devices. As can be seen from Equations (3) and (4), zinc(II) sulfide leaching requires the addition of dissolved oxygen, whereas zinc(II) oxide does not. In addition, as studies of the leaching of Zn(II) from sphalerite in an acidic environment have shown, elemental sulfur [39,40,41,42,43,44,45], as well as other insoluble salts (lead(II) sulfate(VI) and barium sulfate(VI)), may form on the surface of zinc(II) sulphide, hindering further leaching [39,46,47].

ZnO + H_2_SO_4_ → ZnSO_4_ + H_2_O (3)

2 ZnS + 2 H_2_SO_4_ + O_2_ → 2 ZnSO_4_ + 2 S + 2 H_2_O (4)

Due to this difficult mechanism, the leaching of Zn(II) may also be affected by: changes in the S/L ratio, acid concentration or leaching temperature. Thus, the degree of leaching of Zn(II) is limited by zinc(II) sulphide, which is easily covered with a passive layer of sulfur in sulfuric(VI) acid. In works [48,49,50], using sulfuric(VI) acid of various concentrations for Zn(II) leaching, it was found that the extraction of Zn(II) increases from 70 to 95% with the acid concentration to about 5 M H_2_SO_4_, and then it remains constant at 95% or decreases to 80% with a further increase in H_2_SO_4_ acid concentration (above 5 M). The increase in Zn(II) extraction with an increase in acid concentration from 2 to 5 M and then a decrease with a further increase in H_2_SO_4_ acid concentration may also be caused by the “competition” of other metal ions (Fe(III)) at high acidity, leading to an increase in solution viscosity, which, consequently, inhibits the leaching process of Zn(II) [51].

Figure 2d shows the change in the leaching efficiency of Ni(II) over time. A slight increase in leaching of Ni(II) was observed for most of the considered cases of the used acids and temperatures. The exception is 5 M H_2_SO_4_ (353 K), in which the highest degree of leaching of Ni(II) was obtained, amounting to 75%. For the case of 2 M H_2_SO_4_ and the temperature range of 313–353 K, the degree of Ni(II) leaching did not exceed 20%. In the work [52], a similar result of leaching of Ni(II) from PCBs with the use of 2 M H_2_SO_4_ was obtained, amounting to approx. 13%. It can therefore be assumed that the increase in the concentration of the acid used (5 M), but also the accompanying high temperature (353 K), favors the leaching of Ni(II).

The degree of leaching of Pb(II) remained at a low level, as in the case of Cu(II), not exceeding 1%. The authors of papers [26,53,54,55] also indicate that sulfuric(VI) acid is not conducive to Pb(II) leaching. Lead dissolves poorly in diluted H_2_SO_4_ but dissolves well in concentrated, hot sulfuric(VI) acid [55]. During the reaction of lead with sulfuric(VI) acid, a low soluble compound is formed, which is lead(II) sulphate(VI) (Equation (5)) [26,55]. A passivation layer of lead(II) sulphate(VI) is formed on the lead surface, which effectively inhibits further dissolution of this metal [26]. The formation of lead(II) sulfate(VI) takes place with and without the addition of an oxidizing agent to the acid, as presented in the literature [26,53].

(5) $${Pb}^{2+}+{SO}_{4}^{2-}\to Pb{SO}_{4}$$

With the increase in the concentration of the sulfuric(VI) acid used, the degree of leaching of only Fe(III) and Ni(II) increased, while in the case of other metal ions (Cu(II), Sn(IV), Zn(II), Pb(II)), their leaching efficiency did not increase.

#### Influence of Temperature

**Figure 3:**
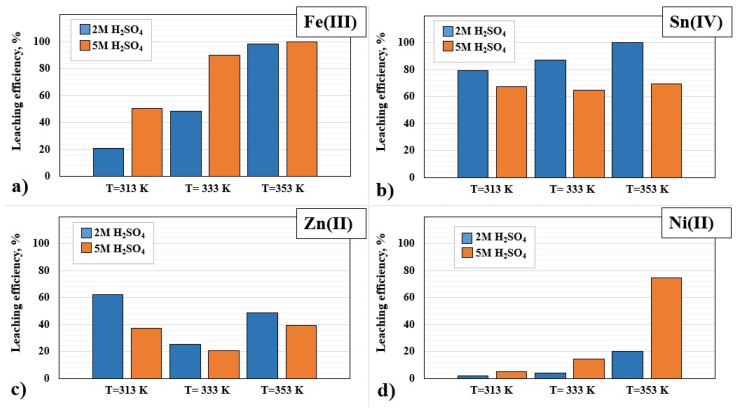
Dependence of the leaching efficiency of: (**a**) Fe(III), (**b**) Sn(IV), (**c**) Zn(II), (**d**) Ni(II) on temperatures of 313, 333, 353 K for 2 and 5 M H_2_SO_4_ (leaching time: 480 min).

Figure 3a presents the results of Fe(III) leaching with a solution of 2 and 5 M H_2_SO_4_, which indicate that the process determining factor influencing the increase in Fe(III) leaching is the temperature, the increase of which is conducive to the transfer of Fe to the solution. The highest leaching efficiency of Fe(III) was obtained at the temperature of 353 K by leaching 2 M H_2_SO_4_ (98%) and 5 M H_2_SO_4_ (100%). Therefore, the temperature of 353 K was selected for the 1st stage of the main research as a factor for effective leaching of Fe(III) from the PCBs.

Similarly, in the case of Sn(IV) (Figure 3b), the significant effect of temperature on the leaching efficiency of tin(IV) using 2 M H_2_SO_4_ (79% at 313 K, 86% at 333 K and 100% at 353 K) is clearly visible. Meanwhile the degree of leaching of Sn(IV) with 5 M H_2_SO_4_ was as follows: 67% at 313 K, 64% at 333 K and 69% at 353 K. Literature reports indicate a weak solution of Sn in H_2_SO_4_ solutions, not exceeding 22% [31,35,36]. However, in the work [56], the use of 1 M H_2_SO_4_, S/L = 1/10 g/cm^3^ and increasing the temperature to 328 K resulted in a visible increase in the Sn(IV) leaching rate of 26% within 90 min. In turn, in the work [36], 26% Sn(IV) was also achieved using 2 M H_2_SO_4_ and 30% H_2_O_2_ at a temperature of 323 K within 180 min. Therefore, the temperature has a significant impact on the efficiency of the Sn dissolution reaction in H_2_SO_4_. Under the given leaching conditions (2 M H_2_SO_4_, S/L = 1/10 g/cm^3^, 353 K, 480 min), the tin could not be passivated or oxidized only to a small extent, hence the high results of dissolution of this metal in sulfuric(IV) acid. In addition, the amount of dissolved oxygen present in the leaching solution is a major factor in determining whether the reaction of tin with sulfuric(VI) acid will form soluble Sn(IV) sulfate or insoluble Sn(IV) hydroxide since the growth oxygen concentration is directly favorable for the formation of tin(IV) hydroxide [39].

The increase in temperature was not conducive to the leaching of Zn(II) (Figure 3c). The highest leaching efficiency of Zn(II) with a solution of 2 M H_2_SO_4_ was obtained at the lowest process temperature (313 K), amounting to 62%. In the case of 5 M H_2_SO_4_ between the temperatures of 313 K and 353 K, the leaching efficiency of Zn(II) was about 40%. A similar result was obtained in the work Silvas et al. [31], where by leaching with 1 M H_2_SO_4_ at a temperature of approx. 353 K, the leaching efficiency of Zn(II) was 40%. The lowest level of leaching of zinc(II) with solutions of 2 and 5 M H_2_SO_4_ was achieved at the temperature of 333 K, amounting to 25% and 20%, respectively. Therefore, the set temperature of 333 K is the least effective for the Zn(II) leaching process. As mentioned earlier, when leaching Zn(II) with sulfuric(VI) acid of various concentrations, this is most likely due to the consumption of all the zinc(II) oxide (Equation (3)), leaving only zinc(II) sulphide, which is covered with a passive layer of sulfur (Equation (4)), reducing the leaching of Zn(II). In addition, in works [48,49,50,57,58], where metallurgical dusts, ores and zinc concentrate were studied, the test results indicate that the rate of zinc dissolution slightly increased with increasing temperature. It was found that with the use of H_2_SO_4_ and a different range of leaching temperatures, the temperature had no significant effect on the extraction of Zn(II).

Figure 3d shows the results of the leaching efficiency of Ni(II) using 2 and 5 M H_2_SO_4_. Within the range of applied temperatures, the value of the leached Ni(II) increases with the increase in temperature. Using 2 M H_2_SO_4_, approximately 2, 4 and 20% leaching of Ni(II) at temperatures of 313, 333 and 353 K was obtained, respectively. Using 5 M H_2_SO_4_, the leaching results increased, respectively: 5, 14 and 75% at temperatures of 313, 333 and 353 K.

The degree of Pb(II) leaching, both with 2 M H_2_SO_4_ and 5 M H_2_SO_4_, remained at a low level and did not exceed 1%, regardless of the temperature used. Due to the low level of Pb(II) leaching, the results are also not shown in the figure.

The degree of leaching of metal ions such as Fe(III), Sn(IV), Ni(II) significantly increased with increasing temperature. Increasing the temperature did not increase the Cu(II), Zn(II) and Pb(II) leaching efficiency.

### 3.2. Leaching with 2 M H_2_SO_4_ Solution (1st Stage)

**Figure 4:**
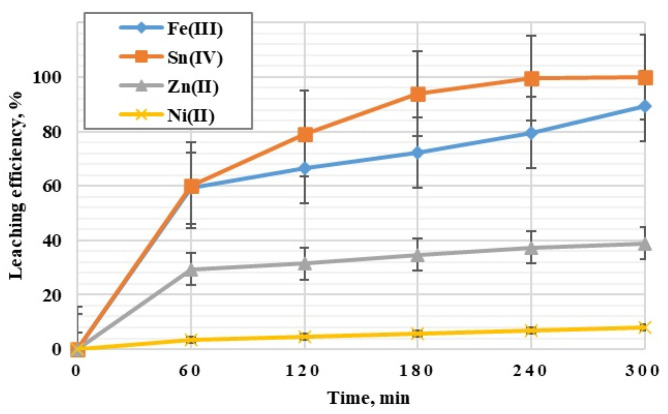
Leaching efficiency of Fe(III), Sn(IV), Zn(II), Ni(II) from PCBs with 2 M H_2_SO_4_ (temperature 353 K, leaching time: 300 min).

Among the experiments using 2 and 5 M H_2_SO_4_, two-stage leaching (1st stage), 2 M H_2_SO_4_ and a temperature of 353 K were selected for further main research. The indicated conditions at S/L = 1/10 g/cm^3^ and leaching time of 300 min ensure leaching of Fe(III) at 98% and Sn(IV) at 100% from PCBs, assuming that the remaining metal ions, especially Cu(II), will be leaching in the next—2nd stage—of research with the use of an additional leaching agent, which is HNO_3_, and oxidants: H_2_O_2_ and O_3_. The solutions used in the research will be reused. As part of the 1st stage of the main research, five leaching tests were performed under identical experimental conditions. Figure 4 presents the obtained results of the leaching of metal ions (Fe(III), Sn(IV), Zn(II), Ni(II)).

The degree of leaching of Cu(II) did not exceed 1%. Therefore, it was confirmed that the adopted target of Cu(II) leaching will be achieved in the next—2nd—stage, using HNO_3_ as the leaching agent, with the addition of oxidants, which will increase the concentration of Cu(II) in the solution.

Figure 4a shows the results of Fe(III) leaching efficiency. There is a common trend of intensive increase in Fe(III) leaching efficiency during the first 60 min of leaching, remaining in the range of 52–66%. Over the next fewvhours, there is a gradual increase in Fe(III) leaching efficiency in all samples. In the final phase of the process, after 300 min, the percentage of Fe(III) leaching was obtained for each of the five samples in the range of 86–94%, of which the highest value of 94% was obtained for sample 5, while the lowest leaching efficiency of Fe(III)—86%—was obtained for sample 2.

The test results presented in Figure 4b showed that the leaching efficiency of Sn(IV) increases over time, and the efficiency is high in the adopted experimental conditions. Already after 60 min of the experiment, the leaching efficiency of Sn(IV) was obtained at 52–66%. After another two hours, the leaching efficiency of Sn(IV) was above 90%. The complete leaching of Sn(IV) was achieved after 300 min of testing in all samples.

Figure 4c shows the change in the degree of leaching of Zn(II) over time. During the first hour of testing, the Zn(II) leaching efficiency was reached, ranging from 25–32% for five samples. After 120 min, this range increased slightly to 27–34%. The lowest leaching efficiency of Zn(II) was obtained for test 2, amounting to 32% (300 min). The highest efficiency was obtained for test 1, where 45% of Zn(II) was leached within 300 min.

Figure 4d shows a common trend of increasing Ni(II) leaching efficiency during the first 60 min of leaching, not exceeding 5%. Over the following few hours, there is a slight increase in all samples ranging from 4 to 9%. The lowest Ni(II) leaching efficiency, lasting from 120 to 300 min of the experiment, was obtained for sample 2 (7%). However, from 180 min to the end of the study, the highest increase is visible for sample 1, amounting to approx. 9%.

The leaching efficiency of Pb(II) did not exceed 2%. The difficulties associated with leaching Pb with sulfuric(VI) acid result from the formation of PbSO_4_, which, as a sparingly soluble compound, covers the metal surface, inhibiting further Pb dissolution. Lead is well dissolved by HNO_3_, forming soluble lead(II) nitrate(V):

(6) $$Pb+2HNO_{3}\to Pb(NO_{3}{)}_{2}+H_{2}.$$

The increase in the Pb(II) leaching efficiency was achieved in the second stage of research using nitric(V) acid, whose high efficiency of Pb dissolution was described in the literature [18,19,59].

The leaching efficiency of all metal ions increases with time; however, for Fe(III), Sn(IV) and Ni(II), the efficiency is high under the experimental conditions.

For the selected, significant from the point of view of metal ion studies of Fe(III) and Sn(IV), a statistical analysis of selected leaching degree results from the first stage of the research was carried out. In order to summarize the data set and draw some basic conclusions and generalizations about the set, the coefficient of variation and range were calculated for all Fe(III) and Sn(IV) samples (for each hour). The significance level of α = 0.05 was assumed. The calculated coefficient of variation for the Fe(III) set ranges from 0.03 (for 5 h) to 0.10 (for 4 h). The range of results is from 7.40 to 14.90. On the other hand, for the Sn(IV) set, the coefficient of variation ranges from 0 (for 5 h) to 0.0613 (for 1 h). The range of the results is from 0 to 8.73.

**Figure 5:**
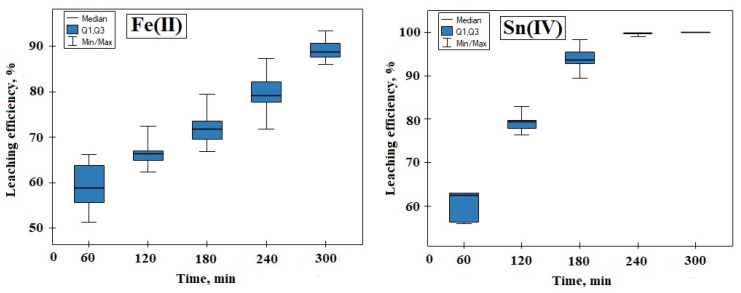
Leaching efficiency as a function of time for: Fe(II) and Sn(IV) (temperature 353 K, leaching solution: 2 M H_2_SO_4_).

Then, a normality test was performed to check the normality of the distribution of variables for the Fe(III) and Sn(IV) sets. For this, the Shapiro–Wilk test was chosen. The results of the test indicate a non-normal distribution for both Fe(III) and Sn(IV) (α = 0.05, *p* > α); therefore, it was decided to perform the Kruskal–Wallis ANOVA test in the next step. This test does not assume a normal distribution. The results of the Kruskal–Wallis test indicate statistically significant differences in the set (*p* = 0.0004 for Fe(III) and *p* = 0.0001 Sn(IV)); therefore, another POST-HOC ranked test was performed to determine the differences between individual pairs. Based on the value of the *p* parameter, statistically significant differences were found between the results of Fe(III) and Sn(IV) leaching at 1, 2, 4 and 5 h. In the next step, the Jonckheere–Terpstra trend test was performed. The results obtained for Fe(III) and Sn(IV) (*p* < 0.05) indicate the presence of an increasing trend as a function of time (Figure 5).

Therefore, Pearson’s r test was performed. Pearson’s r was determined to test whether there is a linear relationship. Based on the test result, the *p* < α value was determined, which confirms the above-mentioned relationship.

**Figure 6:**
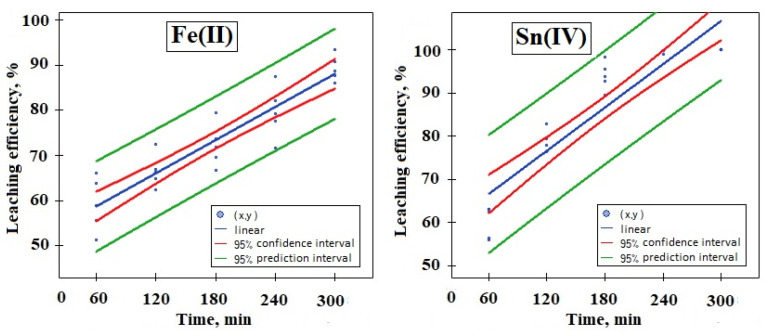
Linear model for: Fe(II) and Sn(IV) (leaching solution: 2 M H_2_SO_4_, leaching time: 300 min).

After carrying out the analysis of the residuals, the value of the coefficient r = 0.920 (r^2^ = 0.847) was obtained. Similarly, in the case of the Fe(III) results, the value of the coefficient r = 0.921 (r^2^ = 0.848) was obtained after analyzing the residuals. The linear model for both cases is also shown graphically in Figure 6. The results obtained from the statistical analysis confirm a slight difference between the obtained results of Fe(III) and Sn(IV) leaching with H_2_SO_4_ solution. It can be assumed that these processes are repeatable.

### 3.3. Leaching with HNO_3_ Solution with the Addition of Oxidants—H_2_O_2_ and O_3_ (2nd Stage)

The residue of the PCB fraction obtained after the first stage of the research was subjected to a further leaching process (2nd stage). For this stage of research, 2 M HNO_3_ and the addition of oxidants H_2_O_2_ and O_3_ were selected.

In acidic solutions, H_2_O_2_ is one of the strongest oxidants [60,61]. Hydrogen peroxide is also a safe and effective oxidant used for PCB leaching, as the end products of decomposition are water and oxygen [53]:

2H_2_O_2_ → 2H_2_O + O_2_. (7)

However, research using O_3_ as an oxidant to leach metal ions from PCBs, which is an equally effective oxidant, has not been used so far. It was used, e.g., in order to increase the efficiency of leaching Cu from chalcopyrite [62,63,64], where the highest efficiency of Cu(II) leaching was obtained when leaching was carried out at ambient temperature (about 296 K) [62]. In the case of ozone, the advantage is also the formation of oxygen as the only reaction by-product during leaching [61,63]:

O_3_ +2H^+^ + 2e^−^ → O_2_ + H_2_O. (8)

As shown in the section on PCB leaching with sulfuric(VI) acid, higher temperature affects leaching efficiency of most of the tested metal ions (Fe(III), Sn(IV) and Ni(II)), intensifying their extraction. However, our own research [65] confirms obtaining high results of Cu(II) recovery during leaching at ambient temperature in the presence of HNO_3_ acid. It was also observed that when the addition of oxidants (H_2_O_2_ and O_3_) was used, the increase in temperature was not conducive to the leaching of metal ions [36,64,66,67]. Therefore, the second stage of leaching tests with the 2 M HNO_3_ and oxidants was carried out at ambient temperature.

For the leaching efficiency of Fe(III), Zn(II) and Ni(II) after 300 min, 100% leaching efficiency of all tested metal ions was obtained in all samples, using both HNO_3_ alone and HNO_3_ with the addition of H_2_O_2_ and O_3_. In total, 100% degree of leaching of Fe(III), Zn(II) and Ni(II) was maintained from the beginning to the end of the experiment (60–300 min).

In the case of zinc, nitric(V) acid dissolved the passive layer of sulfur (formed during leaching with H_2_SO_4_ solution), resulting in 100% leaching of Zn(II) at 298 K. Leaching of zinc(II) oxide and zinc(II) sulphide in nitric(V) acid is shown in Equations (9) and (10) [39]:

ZnO + 2 HNO_3_ → Zn(NO_3_)_2_ + H_2_O (9)

3 ZnS + 8 HNO_3_ → 3 Zn(NO_3_)_2_ + 2 NO + 3 S + 4 H_2_O. (10)

Although the reactions of zinc(II) in sulfuric(VI) and nitric(V) acid are analogous, the main difference lies in the nature of the leaching product. Nitric(V) acid has a beneficial effect on Zn(II) leaching.

**Figure 7:**
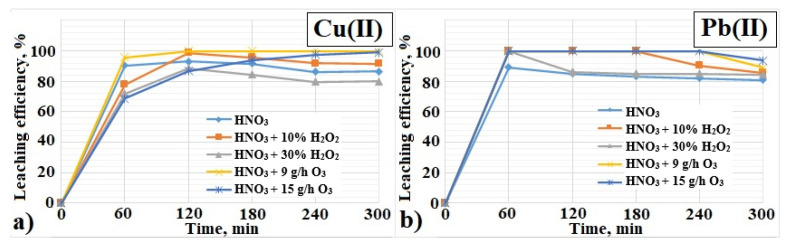
Influence of the oxidizing agent H_2_O_2_ and O_3_ on the leaching efficiency of: (**a**) Cu(II), (**b**) Pb(II) from PCBs (temperature: 313 K, time: 300 min).

Figure 7a shows the influence of the oxidizing agent H_2_O_2_ and O_3_ on the leaching efficiency of Cu(II) from PCBs during 300 min. Within the first 60 min, a rapid increase in the Cu(II) leaching efficiency with the use of 2 M HNO_3_ and oxidants is visible. Using HNO_3_ and 9 g/h O_3_, the Cu(II) leaching efficiency was 96%, and the next 60 min allowed for 100% Cu(II). When 2 M HNO_3_ with the addition of 15 g/h O_3_ was used, 70% of Cu(II) was leached during 60 min, and the Cu(II) leaching efficiency increased over the next few hours, reaching 99% after 300 min of leaching. The least favorable conditions for Cu(II) leaching were observed using 2 M HNO_3_ and 2 M HNO_3_ with the addition of H_2_O_2_. In the initial phase of leaching, the percentage of Cu(II) leaching increased over time, reaching, respectively, after 120 min for HNO_3_, HNO_3_+10% H_2_O_2_ and HNO_3_+30% H_2_O_2_: 93, 99, 89%. During the next 60 min, a slight decrease in the degree of Cu(II) leaching was observed (within the margin of error—5%), and in the next 120 min, the results were 86%, 92% and 80%, respectively. Nitric(V) acid is an effective Cu(II) leaching medium, which is confirmed in publications [19,36,43,59,65]; however, there is no information on the use of this leaching agent with the addition of oxidants in the form of O_3_.

Higher temperatures accelerate the decomposition of hydrogen peroxide and subsequently lead to reduced leaching efficiency [36,66,67,68]. The decomposition of hydrogen peroxide causes the release of oxygen, which is absorbed on the surface of the particles, making it difficult for the particles to contact the peroxide [36]. A higher Cu(II) and Fe(III) leaching efficiency was achieved by adding H_2_O_2_ in one or two portions, compared to the addition of this oxidant several times and in small amounts [36]. The leaching of metals with hydrogen peroxide is also affected by the agitation rate of the system. Degradation of H_2_O_2_ is observed at high stirring speed. In the work [26], an increase in copper leaching was observed by increasing the speed from 200 to 500 rpm, but the decrease in leaching was already visible at the speed of 600 rpm. This difference can be attributed to the accelerated hydrogen peroxide decomposition at higher mixing speeds.

The results presented in Figure 7b indicate high leaching of Pb(II) with the use of 2 M HNO_3_ and oxidants (H_2_O_2_ and O_3_). In this case, 100% of Pb(II) was achieved after one hour. Only when nitric(V) acid was used alone, 89% of Pb(II) was obtained. The further course of the leaching curves is characterized by a decrease in the efficiency of Pb(II) transferred to the solution, recorded at various moments of the leaching process. After 300 min, the following Pb(II) leaching efficiencies were obtained: 80% (2 M HNO_3_), 86% (2 M HNO_3_+10% H_2_O_2_), 85% (2 M HNO_3_+30% H_2_O_2_) and 93% (2 M HNO_3_+O_3_).

The high degree of leaching of Pb(II) with the use of HNO_3_ is confirmed by literature reports [18,19,59]. In the paper [69], the authors Rabah M. et al. achieved complete leaching of Pb from PCBs using 4 M HNO_3_ at 373 K in 60 min. On the other hand, in the works of Kumari A. et al. and Yang J.G. et al. [70,71], HNO_3_ was used to remove solder from PCBs, achieving 100% efficiency while maintaining the temperature of 298 K for 180 min [71].

## 4. Summary and Conclusions

**Figure 8:**
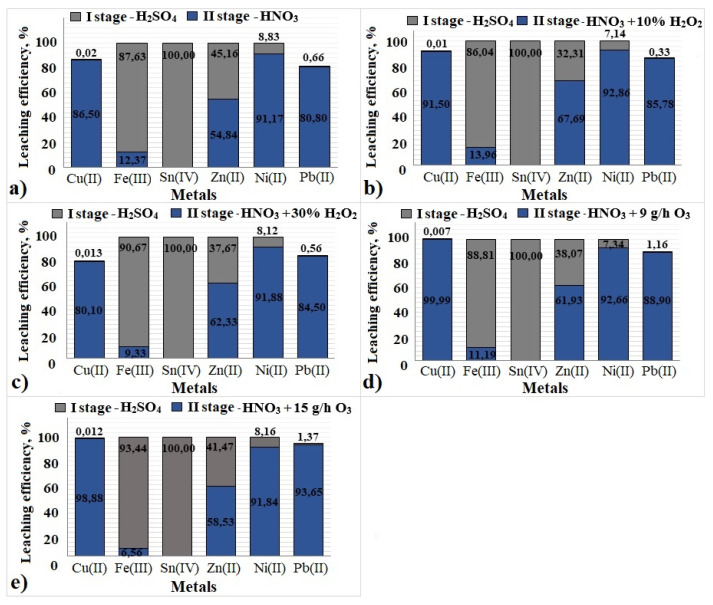
Degree of leaching of metal ions after two-stage leaching process ((■)—1st stage—2 M H_2_SO_4_, (■)—2nd stage—(**a**) 2 M HNO_3_, (**b**) 2 M HNO_3_+10% H_2_O_2_, (**c**) 2 M HNO_3_+30% H_2_O_2_, (**d**) 2 M HNO_3_+9 g/h O_3_, (**e**) 2 M HNO_3_+15 g/h O_3_).

Figure 8 shows the results of two-stage leaching of Cu(II), Fe(III), Sn(IV), Zn(II), Ni(II) and Pb(II) from PCBs. From the solid samples of PCBs 1–5, 100% Fe(III), 100% Sn(IV), 100% Zn(II) and 100% Ni(II) were present in the leached solutions, while Cu(II) was 80–100% (complete leaching of Cu(II) was obtained using O_3_) and 80–95% of Pb(II). Table 2 shows the analysis of studied metals at each stage of the process.

The use of two stages of leaching allows for maintaining the selectivity, separation and recovery of metals: in the first stage of research, Fe(III) and Sn(IV), and in the second stage, the remaining metal ions tested (Cu(II), Zn(II), Ni(II) and Pb(II)).

Removal of Fe(III) by hydrometallurgy from the PCB material at the beginning of the process eliminates the need for using physical magnetic methods, the purpose of which is to separate the magnetic particles of metals (ferrous) from non-magnetic (non-ferrous) particles. There are some problems with PCB magnetic separation. One of them is the agglomeration of the particles, which causes the attraction of some non-ferrous fraction attached to the iron fraction. This leads to the low efficiency of this method. In addition, magnetic separators are ineffective in the case of crushed PCBs. The main challenge with this separation method is the potential for valuable precious metals to be lost from the PCBs. From an economic point of view, these treatments can be associated with high operating costs.

The advantage of the presented hydrometallurgical separation of Fe from other metals by leaching with sulfuric(VI) acid is the avoidance of metal losses in the waste, but also the lack of additional costs associated with the purchase of magnetic separation equipment and energy consumption for its supply during the process, and thus the same high operating costs.

The use of elevated temperature in the tests resulted in an increase in the concentration of Fe(III), Ni(II) and Sn(IV) in the solution. Therefore, for the main tests of the first stage, the temperature of 353 K was used as a factor for the effective leaching of iron, nickel and tin from the PCB material. However, for the second stage, ambient temperature and HNO_3_ and the presence of oxidants (H_2_O_2_ and O_3_) were used due to the fact that the highest efficiency of ozone and hydrogen peroxide was obtained at 298 K. Currently, rising electricity prices result in higher costs for energy utilities. Therefore, the advantage of this process is low energy consumption, which allows for the reduction of the generation of additional costs related to heating and maintaining the temperature throughout the leaching process. The increase in costs is another motivating factor in looking for ways to reduce electricity consumption during hydrometallurgical processes. Saving electricity has an economic dimension but also an ecological one—lower energy consumption means less environmental pollution, less exhaust fumes and greenhouse gases.

The acids used in the research, 2 M H_2_SO_4_ and 2 M HNO_3_, are suitable leaching agents for the recovery of metals from PCBs due to a number of advantages: selectivity, low concentration, low cost and availability, easy regeneration and reuse (HNO_3_), and in a closed system, possibly preventing NO_x_ emissions. Hydrogen peroxide, as an oxidant, is environmentally friendly, as the end products of its decomposition are oxygen and water. Similarly, ozone, used as an oxidant, can be a reasonable alternative, also providing environmental benefits in the leaching process mainly due to the formation of oxygen as the only by-product of the reaction.

Printed circuit boards are a multi-component material, which includes about 40 metals. The research focused on the recovery of iron, copper, tin, zinc, nickel and lead due to their high concentration in the boards and the increasing environmental pressure for the recovery of these metals (mainly Pb, Ni) or economic reasons (Cu, Fe, Sn, Zn). The choice of the hydrometallurgical method was dictated by environmental conditions (no dust or sediments generated, lower amount of energy used) and economic conditions (also lower amount of energy used).

The Fe(III) leaching efficiency in the first stage of the research was <98% with the use of 2 M H_2_SO_4_ and the temperature of 353 K, which made it possible to omit the stage of magnetic separation of the PCB fraction at the beginning of the research. Additionally, in the same stage, 100% of Sn(IV), about 50% of Zn(II) and 20% of Ni were leached.

A clear intensification of the metal ion leaching process, especially Cu(II) and Pb(II), occurs in the 2nd stage of the research, with the use of HNO_3_ and the addition of ozone. Ozone was first used as an additional oxidant to nitric(V) acid. Increasing the O_3_ flow to 15 g/h increases the leaching rate of Pb(II) (94%), while lowering it to 9 g/h increases the leaching rate of Cu(II) (99%). The remaining metal ions (Fe(III), Sn(IV), Zn(II), Ni(II)) both without and with the addition of oxidants (H_2_O_2_ and O_3_) remain leached at a constant level (100%). Research is carried out at the stage of separation of metal ions from the solution from the first and second stages. The next step in the research will be the separation of all tested metals (Fe, Cu, Sn, Zn, Ni and Pb).

In conclusion, the highest leaching efficiency of all tested metal ions was obtained for the variant:

- 1st stage of leaching—2 M H_2_SO_4_, temperature 353 K;
- 2nd stage of leaching—2 M HNO_3_, 9 g/h O_3_, temperature 298 K, which allowed for the leaching of Fe(III), Cu(II), Sn(IV), Zn(II), Ni(II) in 100% and Pb(II) in 90%.

## Figures and Tables

**Table 1:** List of studies on the recovery of metals from PCBs using acids [17,18,19,20,21,22,23,24,25,26,27,28,29,30,31,32,33].

Leaching Agent	Recovery of Metals	Ref.
1–6 M HNO_3_	99% Cu, 70% Sn, 99% Pb	[18]
2–5 M HNO_3_	99% Cu, 68% Ag	[19]
0.5–3 M HNO_3_	53–92% Cu, 13–65% Pb, 94–99% Fe	[20]
1–6 M HCl, 1 M (HNO_3_, H_2_SO_4_, C_2_H_4_O_2_ C_6_H_8_O); 10 M NaOH	100% Cu, Zn, Sn, Ni, Pb, Fe	[21]
0.2–2 M HCl	100% Sn, 4.5% Cu	[22]
1–4 M HCl + Cl_2_	71% Cu, 98% Zn, 96% Sn, 96% Pb	[23]
HCl + FeCl_3_	75% Cu	[24]
H_2_SO_4_	48% Cu, 55% Al, 45.5% Zn, 61% Ni	[25]
1.2 M H_2_SO_4_ + 10% H_2_O_2_	75% Cu	[26]
2 M H_2_SO_4_ + 5% (30%) H_2_O_2_	90% Cu	[27]
2.5 M H_2_SO_4_ + 20% H_2_O_2_ + CO_2_	89% Cu	[28]
1 M H_2_SO_4_ + FeSO_4_	97% Cu	[29]
2 M H_2_SO_4_ + 20% H_2_O_2_; 3 M H_2_SO_4_ + 20% H_2_O_2_	81% Cu	[30]
1 M H_2_SO_4_ I stage; 1 M H_2_SO_4_ + H_2_O_2_ II stage	90% Al, 8.6% Sn, 40% Zn (I stage),	[31]
	100% Cu, 60% Zn, 10% Al (II stage)	
I stage: 2 M H_2_SO_4_ + 35% H_2_O_2_	85% Cu (I stage),	[32]
II stage: 2 M H_2_SO_4_ + 35% H_2_O_2_	14% Cu (II stage); total: 99% Cu	
Hydrogen sulfate ionic liquids 0.5 M + H_2_O_2_	30% Cu	[33]

**Table 2:** Analysis of metal content in samples before leaching and after the 1st and 2nd stage of leaching for sample 4 with best variant of experiments.

Metal		Metal Content in PCB, g					
		Cu	Fe	Sn	Zn	Ni	Pb
Initial mass of feed, g		8.000	0.725	0.555	0.077	0.225	0.082
After 1st stage of leaching H_2_SO_4_	Mass in the solution, g	0.058	0.652	0.555	0.031	0.016	0.002
	Leaching efficiency	0.73%	89.93%	100%	40.26%	7.12%	2.43%
After 2nd stage of leaching HNO_3_ with 9 g/h O_3_	Mass in the solution, g	7.942	0.073	0	0.046	0.209	0.080
	Leaching efficiency	100%	100%	0%	100%	100%	90%
Mass of the metal in PCB after two stages of leaching, g		0	0	0	0	0	0.008

## Data Availability

Data on request.
